# A Context‐Informed Evolutionary Concept Analysis of *Ikigai* in Later Life: Evidence Relevant to Older People in Korea

**DOI:** 10.1111/opn.70092

**Published:** 2026-07-03

**Authors:** HeeKyung Chang, JuHee Seo, Minji Park, Youngjoo Do

**Affiliations:** ^1^ College of Nursing Gyeongsang National University Jinju Gyeongsangnam‐do South Korea

**Keywords:** concept analysis, gerontological nursing, ikigai, Korea, meaning in life, older people

## Abstract

**Aims and Objectives:**

To clarify how *Ikigai* and closely related meaning constructs are described in literature relevant to older people in Korea and to propose a provisional, context‐informed conceptual framework for gerontological nursing.

**Background:**

*Ikigai*, often glossed as “a life worth living,” has been associated with well‐being in later life, yet its meaning and operationalisation vary across settings. Greater conceptual clarity is needed to support culturally responsive nursing assessment and care planning.

**Methods:**

Rodgers' evolutionary method of concept analysis (Rodgers, 2000) was used to examine contemporary scholarly use of *Ikigai* and related concepts in 13 peer‐reviewed studies (published 2002–2024; searched January 2000–June 2025), of which 10 were conducted outside Korea and three in Korea. Data were analysed to identify defining attributes, antecedent contexts, consequences and related concepts.

**Results:**

Six defining attributes were identified: psychological equanimity, purposefulness in life, self‐worth and personal value, social connectedness, cultural belonging, and reflective wisdom and self‐integration. Four antecedent contexts were identified: family and intergenerational change, cultural and value transformation, health and functional challenges, and existential and social disconnection. Four consequence domains were identified: emotional stability and psychological balance, active health orientation and functional preservation, life fulfilment and satisfaction, and community integration and social engagement. In literature relevant to older people in Korea, *Ikigai* was not presented simply as a list of valued sources, but was provisionally interpreted as a process through which relational sources such as family roles, intergenerational continuity and everyday responsibilities may be internalised as an enduring sense of life's worth.

**Conclusions:**

*Ikigai* may be understood as a dynamic and context‐dependent meaning process in later life that includes both valued sources of worth and a sense of life's worth. This review offers a provisional conceptual framework for gerontological nursing and supports further qualitative and measurement research in Korea.

**Implications for Practice:**

This framework can support gerontological nurses in assessing meaning, dignity, purpose and relational continuity alongside physical and functional indicators. Nurses may use open‐ended questions and observable indicators to identify valued roles, relationships and routines that sustain older people's sense that life is worth living. Meaning‐centred and culturally responsive care planning may help support participation, autonomy and continuity during later‐life transitions.

## Summary

**What does this research add to existing knowledge in gerontology?:**

- This analysis conceptualises *Ikigai* as a culturally embedded, relational and dynamic meaning‐making process, extending gerontological understanding of purpose beyond primarily individual‐focused frameworks.
- The study delineates defining attributes, antecedent contexts and consequences, strengthening conceptual clarity for a meaning construct that is inconsistently operationalised across later‐life research.
- Findings suggest that, in literature relevant to older people in Korea, relational sources of meaning are not merely identified but are personally integrated into a sense of life's worth.

**What are the implications of this new knowledge for nursing care for and with older adults?:**

- The framework supports nursing assessment of meaning, dignity and purpose alongside physical and functional indicators, enabling more holistic and person‐centred care planning.
- A relational framing helps nurses recognise early indicators of meaning‐related distress, withdrawal or reduced agency that may be overlooked in routine symptom‐focused assessments.
- Nurses can align interventions with key attributes by supporting valued roles, autonomy in daily decisions, relationship continuity and structured life review during care encounters.

**How could the findings be used to influence practice, education, research, and policy?:**

- The findings support the development of meaning‐centred nursing practice, education and research that attends to relational continuity, social participation and culturally responsive communication in later life.
- At the systems level, age‐friendly and supportive care environments may help sustain meaning, reduce isolation and support older people's continued participation in everyday and community life.
- Future qualitative and longitudinal studies should examine how relational sources of meaning are taken up, expressed and supported across later‐life care contexts.

## 1 Introduction

Gerontological nurses support older people to live with dignity, sustain participation and maintain a sense of purpose in later life. This work benefits from concepts that reflect how meaning is made in specific social settings (Reed 2008). In Korea, changes in family structure, work and residence patterns are reshaping how older people experience identity, social participation and meaning in later life (World Health Organization [WHO] 2024; Statistics Korea 2023; Han et al. 2023). As family roles and intergenerational reciprocity change, some older people may renegotiate, sustain or reframe their sources of purpose and connection across later‐life transitions (Kim and Kil 2019; Lee and Hong 2017).

*Ikigai* (生き甲斐), often translated as “a reason for being”, is used in research on well‐being and meaning. In Japanese scholarship, *Ikigai* can refer to sources of purpose (e.g., roles, relationships or activities) and/or the felt sense that one's life is worth living; these usages are related but not identical. Studies in Japan link *Ikigai* with lower mortality, better psychological health and slower functional decline (Tanno et al. 2009; Tomioka et al. 2016). Korea and Japan share some historical influences, but Korea's social history and family change follow a different path (Kim and Kil 2019; Lee and Hong 2017). In Korean contexts, authors sometimes use the term *Ikigai* alongside Korean expressions such as salm‐ui uimi (meaning of life) and saeng‐ui boram (life's reward), raising questions about how this borrowed term aligns with local meaning‐related concepts.

Meaning and purpose are well‐established predictors of psychological adaptation and health‐promoting behaviours in nursing science (Aviad and Cohen‐Louck 2021). While *Ikigai* originated in Japan and has been studied extensively within Japanese contexts (Tanno et al. 2009; Tomioka et al. 2016; Fukuzawa and Sugawara 2023; Kawamura 2025), fewer studies examine how the concept is used outside Japan, and Korean empirical studies remain limited (Kim and Kil 2019; Lee and Hong 2017; Chong et al. 2012; Randall et al. 2022, 2023). In Korea, later‐life meaning‐making takes place within a social context shaped by expectations around family responsibility and intergenerational support, alongside broader changes in work, residence and family life (Kim and Kil 2019; Lee and Hong 2017; Statistics Korea 2023). Although *Ikigai* can be relevant across the life course, gerontological nursing often meets older people at points of role transition and health change, when questions about purpose and contribution come to the fore.

Concept analysis provides a structured way to examine how a concept is defined and used, which can support nursing assessment and intervention development. Rodgers' (2000) evolutionary approach treats concepts as dynamic and context‐dependent and therefore fits cross‐cultural work. In this review, we apply Rodgers' method to literature relevant to older people in Korea to describe how authors define and operationalise *Ikigai* and to identify its reported attributes, antecedents and consequences. Because Korean empirical studies on *Ikigai* remain few, we include both Korean and international literature and interpret the findings in relation to the Korean language and sociocultural context. We present the resulting model as a provisional account that can guide practice and be further refined through future research with Korean older people.

Accordingly, this study conducts a context‐informed evolutionary concept analysis of *Ikigai* in literature relevant to older people in Korea. Specifically, we examine how a concept originating in Japan is currently discussed and contextually elaborated within literature relevant to older people in Korea, rather than seeking to establish a distinctly Korean conceptual structure of *Ikigai*. We therefore aim to: (1) describe how the included literature defines and applies *Ikigai* and related meaning constructs; (2) identify the antecedents, defining attributes and consequences associated with *Ikigai* in this body of literature; (3) examine how Korean‐relevant studies illuminate the relational and experiential dimensions of *Ikigai*; and (4) propose a provisional, context‐informed conceptual model to support culturally responsive gerontological nursing assessment, intervention planning and care.

## 2 Methods

### 2.1 Design

We used Rodgers' (2000) evolutionary concept analysis to examine how *Ikigai* has been conceptualised in literature relevant to older people in Korea. We selected this approach because its iterative and inductive process enables clarification of culturally embedded and context‐dependent concepts. Rodgers' evolutionary method recognises that conceptual meanings evolve over time, context and application, making it particularly suitable for examining cross‐cultural nursing phenomena. Although Rodgers' evolutionary approach informed the analytic procedures, this review did not aim to trace the historical evolution of *Ikigai* over time. Rather, it used Rodgers' framework to examine contemporary concept usage, contextual variation, surrogate terms, defining attributes, antecedent contexts and consequences in literature relevant to older people in Korea. We describe the resulting analysis as “context‐informed” to signal that interpretations are provisional and grounded in the reviewed Korean and contextual literature, rather than empirically derived from primary Korean data.

The method comprises six sequential and iterative steps: (1) identifying the concept and its surrogate or related terms; (2) selecting an appropriate realm for data collection; (3) gathering literature relevant to attributes, contextual factors and boundaries; (4) analysing data regarding defining attributes, antecedents, consequences and related concepts; (5) identifying an exemplar of the concept; and (6) deriving theoretical implications and hypotheses for further development.

We adopted this design to explore how the literature has conceptualised and operationalised *Ikigai* as a culturally embedded construct, including how studies distinguish sources of *Ikigai* (activities/roles/objects) from the felt “sense of Ikigai” and how its defining elements can inform gerontological nursing assessment, communication and care planning in the Korean context. Korean‐context interpretations were anchored primarily in Korean empirical literature, while non‐Korean evidence was used for cross‐context interpretation and boundary clarification.

### 2.2 Search Strategy

The search strategy aimed to identify literature on *Ikigai* and closely related expressions relevant to later life, including studies conducted in Korea and studies used for cross‐context interpretation within Rodgers' evolutionary approach. We searched for studies published between January 2000 and June 2025. The search period was set from January 2000 to June 2025 as a pragmatic boundary for examining contemporary scholarly use of *Ikigai* and related later‐life meaning constructs. This timeframe was chosen to focus on recent gerontological, nursing, health and social science literature relevant to contemporary care contexts, rather than on the historical or philological evolution of the term itself.

*International databases included*: PubMed/MEDLINE, CINAHL (Cumulative Index to Nursing and Allied Health Literature), PsycINFO and Scopus.

*Korean databases included*: KISS (Korean Studies Information Service System) and DBpia. Search terms were developed in consultation with research librarians and refined through pilot searches to optimise relevance and sensitivity. Where available, we used controlled vocabulary (e.g., database subject headings) alongside keyword searches.

*English search terms*: (“Ikigai” OR “life worth living” OR “meaning in life” OR “purpose in life”) AND (“older adults” OR “elderly” OR “older people” OR “ageing” OR “aging” OR “later life”) AND (“Korea*” OR “Asian” OR “East Asian”). To capture how *Ikigai* has been operationalised beyond Korea for interpretive comparison, we also ran an additional search without the Korea‐related block.

*Korean search terms*: (“이키가이” OR “생의 의미” OR “삶의 보람” OR “삶의 가치”) AND (“노인” OR “고령자” OR “노년”).

Additional strategies included hand‐searching the reference lists of included studies and targeted manual searching of Korean nursing and social science journals. Citation tracking and expert consultation were used to support search refinement and relevance checks rather than as separate sources of included records. We included peer‐reviewed English‐ and Korean‐language studies and managed records in EndNote, removing duplicates using automated functions and manual checks. To maximise retrieval sensitivity and reproducibility, we retained legacy indexing terms such as “elderly” in the search strategy, although we used non‐ageist terminology (e.g., “older people”) in reporting.

### 2.3 Inclusion and Exclusion Criteria

Studies were eligible if they: (1) were peer‐reviewed empirical research articles published in English or Korean between 2000 and 2025; (2) addressed *Ikigai* explicitly (e.g., “Ikigai/生き甲斐/이키가이”) or clearly positioned “life worth living” as a surrogate expression of *Ikigai*; or examined conceptually related expressions (e.g., meaning in life, purpose in life) as terms used within Rodgers' evolutionary approach to clarify conceptual boundaries, antecedents and consequences of *Ikigai*; (3) reported data relevant to older people (generally ≥ 60 years) or provided extractable findings about later‐life meaning‐making; this included later‐life contexts with extractable conceptual data (e.g., family caregiving for an older parent living with dementia); and (4) included conceptual or experiential descriptions and/or empirical associations that could inform the identification of attributes, antecedents and consequences.

Because Korean empirical literature on *Ikigai* remains limited, we included two complementary bodies of evidence: (a) studies conducted in Korea or involving Korean older people and (b) studies conducted outside Korea that examined *Ikigai* or adjacent concepts of meaning and purpose to support interpretive contrast across contexts within Rodgers' evolutionary approach. We restricted Korea‐specific interpretations to the Korean studies and to evidence with direct relevance to Korean cultural contexts. Studies were excluded if they: (1) were dissertations, theses, essays, narrative reviews or non‐peer‐reviewed materials; (2) addressed generic well‐being constructs without extractable conceptual content relevant to *Ikigai* (e.g., meaning/purpose measured only as a covariate with no interpretive or conceptual linkage to later‐life meaning‐making); or (3) did not include extractable conceptual data relevant to attributes, antecedents, consequences or related concepts.

### 2.4 Study Selection and Data Extraction

**FIGURE 1:**
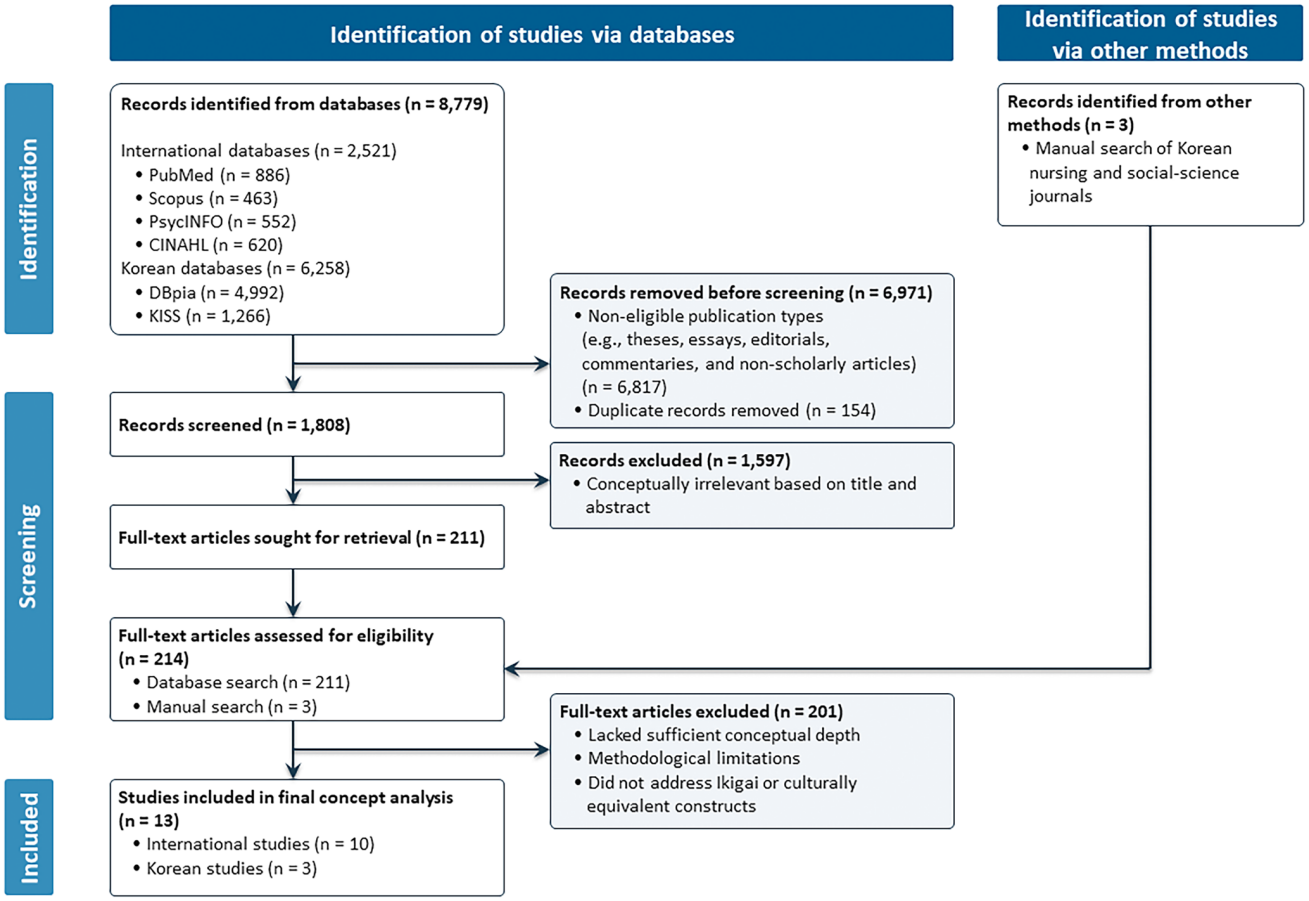
PRISMA flow diagram for study selection in the evolutionary concept analysis of Ikigai in literature relevant to older people in Korea. A total of 8779 records were identified through database searching and three additional records through manual searches. After removal of 6817 non‐eligible publication types and 154 duplicate records, 1808 records remained for title and abstract screening. Of these, 1597 records were excluded as not conceptually relevant, leaving 211 full‐text articles for retrieval through database screening. Together with the three manually identified records, 214 full‐text articles were assessed for eligibility. After full‐text assessment, 201 articles were excluded, and 13 studies were included in the final concept analysis (10 international and 3 Korean).

A total of 8779 records were identified through database searching, and three additional records were identified through manual searches. After removing 6817 non‐eligible publication types (e.g., theses, essays and review articles) and 154 duplicate records, 1808 records remained for title and abstract screening. Of these, 1597 records were excluded as not conceptually relevant, leaving 211 full‐text articles for retrieval through database screening. Together with the three manually identified records, 214 full‐text articles were assessed for eligibility. After full‐text assessment, 201 articles were excluded because they lacked sufficient conceptual relevance, had methodological limitations or did not address *Ikigai* or culturally equivalent constructs. In total, 13 studies were included in the final concept analysis (Figure 1).

Initial screening decisions were independently made by two reviewers, and disagreements were then discussed with two additional reviewers until consensus was reached. Inter‐rater reliability between the two primary reviewers was high (Cohen's *κ* = 0.82; Cohen 1960). Any discrepancies were resolved through discussion and consensus. The overall study selection process followed the PRISMA 2020 guidelines (Page et al. 2021) to ensure transparency and reproducibility.

We extracted data using a standardised concept analysis form developed for this study. Extracted information included study characteristics (author, year, country, design and sample), conceptual definitions, defining attributes, antecedent contexts, consequences and related or surrogate concepts. All data were independently extracted and cross‐checked by the four reviewers until full agreement was reached. A consolidated summary of study populations, target constructs/terms, operationalisations and quality appraisal results for all included studies is provided in Table S1.

### 2.5 Quality Appraisal

We assessed the methodological quality and conceptual adequacy of the included studies. Quality appraisal was conducted by four independent reviewers using criteria adapted from the Joanna Briggs Institute (JBI) Critical Appraisal Tools (JBI 2020) and Whittemore and Knafl's (2005) framework for integrative reviews. Each study was evaluated on:

1. Clarity of research aims and alignment with the *Ikigai* construct;
2. Appropriateness of study design and methodology;
3. Rigour of data collection and analysis procedures;
4. Relevance to the Korean cultural context or applicability to Korean older populations; and
5. Contribution to the conceptual understanding of *Ikigai*.

All 13 included studies demonstrated adequate methodological quality and conceptual relevance for concept analysis purposes. Studies were not excluded based solely on quality scores, as Rodgers' (2000) evolutionary method emphasises capturing the full range of concept usage across diverse contexts and methodologies. Nevertheless, quality considerations informed the interpretation and synthesis of findings, with higher‐quality studies given greater weight in defining the core attributes of *Ikigai*. This consensus‐based approach enhanced the credibility, transparency and reproducibility of the final conceptual model. Each criterion was scored on a 0–2 scale (0 = unclear/weak, 1 = partial, 2 = clear/strong). Per‐study quality appraisal ratings are reported in Table S1.

### 2.6 Data Analysis

We analysed data using Rodgers' (2000) evolutionary concept analysis approach, which provides a systematic and inductive framework for clarifying conceptually complex and culturally embedded nursing phenomena. This approach recognises that conceptual meanings evolve across time, disciplines and sociocultural contexts, making it appropriate for examining *Ikigai* as a dynamic construct in Korea.

Following Rodgers' six iterative steps, four independent researchers collaboratively conducted the analysis to enhance rigour and transparency.

1. *Identification of the concept and surrogate or related terms*: *Ikigai* and its culturally equivalent expressions (e.g., meaning in life, purpose in life, life's reward [삶의 보람]) were identified.
2. *Selection of the setting and sample for data collection*: The final dataset consisted of 13 peer‐reviewed studies (5 studies addressing *Ikigai* or the notion of life worth living and 8 studies addressing conceptually related expressions; 10 international and 3 Korean) that met the inclusion criteria.
3. *Data collection relevant to attributes and contextual basis*: Key conceptual statements, definitions and contextual descriptions were extracted from each study.
4. *Data analysis*: We analysed the extracted data thematically to identify antecedents, defining attributes, consequences and related concepts, and we iteratively compared themes across studies to refine conceptual boundaries. Cross‐checking among the four reviewers ensured analytical rigour.
5. *Identification of exemplars*: Representative excerpts illustrating *Ikigai* in empirical or theoretical contexts were identified to demonstrate the practical relevance of the concept.
6. *Interpretation and implications for further development*: We synthesised the findings to construct a culturally grounded conceptual framework that reflects the dynamic and context‐dependent nature of *Ikigai* in literature relevant to older people in Korea.

Throughout the process, an audit trail and consensus meetings were maintained to ensure methodological rigour, traceability and reproducibility.

To enhance the practical applicability of the conceptual findings, illustrative examples of nursing assessment questions, clinically assessable indicators and corresponding interventions aligned with each defining attribute of *Ikigai* are provided in Table S2.

For Korean‐language articles, a bilingual Korean–English reviewer extracted relevant text in Korean and translated key excerpts into English for coding. To support accuracy and conceptual equivalence, a second bilingual reviewer reviewed translated segments and terminology (including surrogate terms), and discrepancies were resolved through discussion. Illustrative exemplars identified through this step are presented in Section 3.8 and Table S4.

### 2.7 Ethical Considerations

As a secondary analysis of previously published literature, this study did not require institutional review board approval. All included studies were reported to have obtained ethical approval in their original publications.

## 3 Results

Thirteen studies met the inclusion criteria for this evolutionary concept analysis (Figure 1).

The included studies were published between 2002 and 2024, and most (*n* = 11) were published from 2016 onwards. Of these, three studies were conducted in Korea and ten were conducted outside Korea (Japan, *n* = 5; Belgium, *n* = 1; the Netherlands, *n* = 1; China, *n* = 1; Israel, *n* = 1; Turkey, *n* = 1). Table 1 summarises study characteristics.

**TABLE 1:** Characteristics of included studies (*n* = 13).

ID	Author(s), Year	Country	Design/Sample	Study focus/Analytic emphasis	Key concept elements extracted
A1	Watanabe et al. (2024)	Japan	Quantitative (cross‐sectional questionnaire survey), analytic *n* = 6978 (7973 responses), adults aged ≥ 40 years; age‐stratified findings reported for later life	Going‐out frequency, *Ikigai* and mental health	Psychological equanimity; social connectedness; emotional balance
A2	Fukuzawa and Sugawara (2023)	Japan	Quantitative (cross‐sectional survey), *n* = 418, adults aged ≥ 75 years	Loneliness, social support and *Ikigai*	Social connectedness; perceived social roles; community participation
A3	Zhu et al. (2023)	China	Quantitative (cross‐sectional case–control with mediation modelling), total *N* = 992 (LOD *n* = 496; controls *n* = 496), age 60–73 years	Health‐promoting lifestyle, meaning and depression	Health‐promoting motivation; purposefulness; psychological equanimity
A4	Dewitte et al. (2022)	Belgium	Quantitative (longitudinal observational; three annual assessments), *n* = 140 older people living with Alzheimer's disease in nursing homes	Meaning, depression and cognitive functioning	Reflective wisdom; self‐integration; meaning‐related psychological outcomes
A5	Aviad and Cohen‐Louck (2021)	Israel	Quantitative (cross‐sectional survey), *n* = 195, adults aged 65–100 years	Purpose in life and suicide risk	Purposefulness; psychological equanimity
A6	Hupkens et al. (2021)	Netherlands	Qualitative (hermeneutic phenomenological; three interview waves with photo‐elicitation), *n* = 24 community‐dwelling older people receiving home nursing	Meaning in daily life with home nursing	Daily meaning‐making; social connectedness; purpose orientation
A7	Aydın et al. (2020)	Turkey	Quantitative (cross‐sectional), *n* = 144 (71 nursing‐home residents; 73 community‐dwelling older people)	Spiritual well‐being, meaning and mental health	Spiritual well‐being; cultural belonging; existential meaning
A8	Fukuzawa et al. (2019)	Japan	Quantitative (panel longitudinal), baseline *n* = 1068; follow‐up *n* = 686	Social capital, human capital and *Ikigai*	Social connectedness; relational continuity; community engagement
A9	Kim and Kil (2019)	Korea	Qualitative interpretive meta‐integration (13 included qualitative studies)	Meaning of life in Korean later life	Family‐based meaning; cultural belonging; experience and creation of meaning
A10	Mori et al. (2017)	Japan	Quantitative (prospective cohort), *n* = 830 adults aged ≥ 70 years; 11‐year follow‐up	*Ikigai* and incident functional disability	Health orientation; purposefulness; active living
A11	Lee and Hong (2017)	Korea	Scale‐development study: qualitative item generation (*n* = 10) and quantitative psychometric testing (*n* = 371 community‐dwelling older people)	Korean meaning‐in‐life scale development	Self‐worth and personal value; purposefulness; will to live
A12	Chong et al. (2012)	Korea	Quantitative (correlational survey), analysed *n* = 497 (562 collected; 65 excluded)	Family relations, meaning and successful ageing	Family connectedness; cultural belonging; self‐transcendent value
A13	Yamamoto‐Mitani and Wallhagen (2002)	Japan	Qualitative (constant comparative interview study), *n* = 26 women family caregivers (13 daughters; 13 daughters‐in‐law) caring for older parents/parents‐in‐law living with dementia	Caregiving, self‐understanding and *Ikigai*	Self‐integration; caregiving roles and relationships; sources and sense of *Ikigai*

*Note:* Where a study reported both recruitment/responses and a final analytic sample, the analytic sample is used in Table 1, with additional counts retained in parentheses where important for interpretation (e.g., A1, A12). Where studies reported multiple analytic samples across timepoints or phases (e.g., baseline/follow‐up or scale‐development stages), these are reported explicitly (e.g., A8, A11).

The included designs comprised quantitative studies (*n* = 9), qualitative studies (*n* = 2), one qualitative meta‐integration (*n* = 1) and one instrument development study (*n* = 1). Quantitative analytic samples ranged from 140 to 6978 participants, while qualitative studies included 24–26 participants; the qualitative meta‐integration synthesised 13 qualitative studies conducted in Korea. Most studies focused on later life (generally ≥ 60 years), while one large survey included adults aged ≥ 40 years and reported age‐stratified findings relevant to meaning‐making in later life.

Defining attributes, antecedent contexts and consequences are presented in Tables 2, 3, 4. To support transparency, Table S1 provides consolidated study details and quality appraisal results for all included studies (A1–A13), and Table S2 presents practice‐oriented assessment questions, clinically assessable indicators and illustrative nursing interventions aligned with the defining attributes.

**TABLE 2:** Defining attributes of *Ikigai* identified in the included studies (*n* = 13).

Defining attribute	Conceptual description	Representative evidence (Study ID)
Psychological equanimity	Emotional steadiness and adaptive balance associated with a sense that life is worth living.	Korean evidence: Not explicitly identifiable in included Korean studies (A9, A11, A12). Contextual evidence: A1, A3, A5
Purposefulness in life	A sustained sense of direction expressed through valued roles and contributions. In the included Korean evidence (A11), purpose is reflected in the value of life, will to live and personally meaningful roles.	Korean evidence: A11 Contextual evidence: A3, A6
Self‐worth and personal value	Perceived personal value and affirmation of continued significance. In the included Korean evidence (A11), this attribute is reflected in the value of life, personal significance and continued meaning.	Korean evidence: A11 Contextual evidence: Not explicitly identifiable in this dataset.
Social connectedness	Meaning derived from reciprocal relationships and social participation. Across studies, social ties are central; in Korean evidence, family‐based reciprocity is particularly salient.	Korean evidence: A9, A12 Contextual evidence: A2, A6, A8
Cultural belonging	Meaning grounded in culturally embedded values, traditions and intergenerational continuity. This attribute is particularly prominent in Korean qualitative and survey studies (A9, A12).	Korean evidence: A9, A12 Contextual evidence: A7
Reflective wisdom and self‐integration	Integration of life experiences through reflection, acceptance, narrative coherence and self‐transcendent values. Korean evidence links reflective integration to moral maturity and intergenerational continuity.	Korean evidence: A9, A12 Contextual evidence: A4, A6, A13

*Note:* Study IDs correspond to the 13 included studies (see Table 1 and Table S1). ‘Korean evidence’ refers to studies conducted in Korea or involving Korean participants (A9, A11, A12). ‘Contextual evidence’ refers to studies conducted outside Korea included for interpretive comparison across contexts within Rodgers' evolutionary approach. When Korean evidence is listed as “not explicitly identifiable,” the included Korean studies did not contain extractable statements that mapped directly onto that attribute label.

### 3.1 Quality Assessment

We appraised methodological quality and conceptual adequacy using predefined criteria covering five domains (aim–construct alignment, design/methodology, data collection and analysis, Korean‐context relevance and contribution to conceptual understanding). Quality appraisal results for each study are reported in Table S1. Overall appraisal scores ranged from 7/10 to 10/10, and we used these ratings to support interpretive transparency rather than to determine study exclusion.

### 3.2 Theoretical Definition of *Ikigai*

Based on the synthesis of findings from 13 studies, *Ikigai* in literature relevant to older people in Korea refers to a multidimensional, culturally grounded construct that integrates a sense that life is worth living, meaning and purpose. In this analysis, “Ikigai” is treated as a focal concept, while meaning‐in‐life and purpose‐in‐life are treated as related concepts of meaning and purpose that help clarify conceptual boundaries (see Table S3).

In the included Korean evidence (A9, A11, A12), meaning and worth are frequently articulated through family roles, relational responsibility and continuity across generations. The Korean‐relevant literature does not present *Ikigai* or meaning simply as a list of external sources. Rather, it shows how relational sources—such as family, roles and intergenerational continuity—are taken up, given personal significance and carried into later‐life experience as value, meaning and a continued sense that life is worth living. In this sense, Korean‐context evidence highlights not only relational and culturally embedded sources of worth but also the experiential process through which such sources become personally meaningful.

Theoretically, *Ikigai* can therefore be described as including both (a) sources of worth (valued roles, relationships or activities) and (b) a subjective sense that life is worth living. In this article, “process” refers to the conceptual linkage through which antecedent contexts, defining attributes and reported consequences are connected in the included literature, rather than to a historical change in the definition of *Ikigai*. Together, these elements provide a working conceptual foundation for gerontological nursing assessment and care planning, while recognising that Korea‐specific interpretations are anchored primarily in the included Korean studies.

### 3.3 Defining Attributes of *Ikigai*

Analysis of the 13 included studies identified six interrelated defining attributes that characterise how *Ikigai* and closely related concepts of meaning and purpose are described in later life (Table 2). Evidence is reported as Korean evidence (A9, A11, A12) versus contextual evidence (studies conducted outside Korea); where Korean evidence was not explicitly identifiable for an attribute label, we report contextual evidence for interpretive comparison across contexts.

These attributes should be understood as a conceptual synthesis rather than a claim that all attributes are uniquely Korean. In the included Korean evidence, relational and culturally embedded sources of worth are particularly salient. At the same time, the Korean‐relevant literature suggests that such sources are not merely named as external conditions; they are taken up and personally integrated within later‐life experience, thereby contributing to the value of life, meaning and the sense that life is worth living. Several attribute labels, however, draw mainly on broader comparative literature and are therefore presented for boundary clarification rather than as direct Korea‐specific evidence.

#### 3.3.1 Psychological Equanimity

*Ikigai* was described as being associated with emotional steadiness and adaptive balance (Table 2). In the included set, evidence for this attribute came mainly from non‐Korean or adjacent‐construct studies (A1, A3, A5), and Korean evidence was not explicitly identifiable for this attribute label. It should therefore be interpreted as boundary‐clarifying rather than Korea‐specific.

#### 3.3.2 Purposefulness in Life

This attribute reflects a sustained sense of direction expressed through valued roles and contributions. In the included Korean evidence (A11), purpose is reflected in components such as value of life and will to live, while contextual evidence describes purpose in relation to activities and roles (A3, A6).

#### 3.3.3 Self‐Worth and Personal Value

This attribute concerns perceived personal value and affirmation of continued significance. Korean evidence (A11) emphasises self‐worth and the value of life, articulated through preferences, perceived significance and continued personal meaning.

#### 3.3.4 Social Connectedness

This attribute refers to meaning derived from reciprocal relationships and participation. Korean evidence (A9, A12) highlights family reciprocity and relational continuity as salient sources of meaning, complemented by contextual evidence on social support and social capital (A2, A6, A8).

#### 3.3.5 Cultural Belonging

This attribute reflects meaning grounded in culturally embedded values, traditions and intergenerational continuity. Korean evidence (A9, A12) supports cultural belonging as a salient component, with contextual evidence (A7) providing comparative insight into spirituality and cultural values.

#### 3.3.6 Reflective Wisdom and Self‐Integration

This attribute describes integration of life experiences through reflection, acceptance, narrative coherence and self‐transcendent values. Korean evidence (A9, A12) links reflective integration to moral maturity and intergenerational continuity, and contextual qualitative studies (A4, A6, A13) provide further illustrations of life review and self‐integration.

Together, these attributes describe *Ikigai* as a multidimensional construct in later life. Korean‐context salience was most directly supported for social connectedness, cultural belonging, and reflective wisdom/self‐integration in the included Korean studies, whereas other attributes were retained as boundary‐clarifying elements rather than as Korea‐specific findings. Practice‐oriented indicators, assessment prompts and illustrative nursing interventions aligned with these attributes are presented in Table S2.

### 3.4 Antecedent Contexts of *Ikigai*

Analysis identified four interrelated antecedent contexts described in the included literature as situations in which older people may re‐evaluate, maintain, reconstruct or renegotiate *Ikigai* (Table 3). These contexts are presented as antecedent conditions rather than direct causes, consistent with an evolutionary concept analysis approach. In this analysis, antecedent contexts are understood as conditions in which previously valued sources of meaning may be disrupted, sustained or reinterpreted, thereby shaping how this sense of life's worth is maintained or restored in later life.

**TABLE 3:** Antecedent contexts of *Ikigai* in later life identified in the included studies.

Antecedent context	Description (with Korean‐context emphasis)	Representative evidence (Study ID)
Family and intergenerational change	Shifts in family roles, caregiving responsibilities or intergenerational expectations that prompt reconsideration of valued meaning sources and life worth. In Korean studies, meaning‐making is frequently organised around family obligation, reciprocity and continuity across generations.	Korean evidence: A9, A12 Contextual evidence: Not explicitly identifiable in the included non‐Korean studies.
Cultural and value transformation	Societal or cultural shifts influencing how older people interpret responsibility, belonging and purpose. Korean evidence highlights how moral–relational norms and culturally familiar practices can support belonging and continuity amid social change.	Korean evidence: A9, A12 Contextual evidence: A7
Health and functional challenges	Experiences of illness, disability or cognitive decline that reshape engagement and meaning orientation. In the included set, direct Korean empirical evidence for this context was limited; therefore, this interpretation should be read as boundary‐clarifying rather than Korea‐specific.	Korean evidence: Not explicitly identifiable in included Korean studies (A9, A11, A12). Contextual evidence: A4
Existential and social disconnection	Loneliness, reduced participation or perceived loss of social role prompting reflection on purpose.	Korean evidence: Not explicitly identifiable in included Korean studies (A9, A11, A12). Contextual evidence: A2, A8, A13

*Note:* Antecedent contexts describe situations in which people may re‐evaluate, maintain, reconstruct or renegotiate *Ikigai*, rather than serving as direct causes. Evidence is reported as Korean evidence (A9, A11, A12) versus contextual evidence (studies conducted outside Korea).

#### 3.4.1 Family and Intergenerational Change

The included Korean evidence described shifts in family roles, intergenerational expectations and caregiving responsibilities as contexts that prompt reconsideration of later‐life meaning. Korean studies highlighted family obligation, reciprocity and continuity across generations as salient elements of meaning‐making. These studies did not treat family merely as an external source of meaning; rather, they showed how family‐related roles and responsibilities were taken up and personally integrated within later‐life experience, contributing to perceived worth, continuity and a continuing sense of meaning in life.

#### 3.4.2 Cultural and Value Transformation

Several studies described later‐life meaning‐making in relation to cultural values and practices. The included Korean evidence emphasised moral–relational norms and culturally familiar practices that support belonging and continuity amid social change. In this respect, cultural belonging was not simply a background variable but part of the interpretive process through which older people located value, obligation and life meaning within changing social conditions.

#### 3.4.3 Health and Functional Challenges

Studies described illness, disability and cognitive change as contexts that reshape engagement and meaning orientation. Direct Korean empirical evidence for this context was limited in the included set; therefore, this interpretation should be read as boundary‐clarifying rather than Korea‐specific. These findings suggest that health and functional challenges should be understood not only as potential threats to *Ikigai*, but also as contexts in which older people may renegotiate valued roles, autonomy, continuity and a sense of meaning under altered conditions.

#### 3.4.4 Existential and Social Disconnection

Studies described loneliness, reduced participation and perceived loss of social role as contexts prompting reflection on purpose. In the included set, this context was identified mainly through indirect comparative support. Conceptually, this antecedent context is important because it clarifies that *Ikigai* is not reducible to the mere presence of social ties, but concerns how older people interpret connection, role continuity and life value when these are threatened or diminished.

### 3.5 Consequences of *Ikigai*

Analysis identified four consequence domains reported as associated with *Ikigai* or closely related concepts of meaning and purpose (Table 4). These consequences reflect emotional, behavioural, existential and social dimensions of well‐being described in the included literature. In this analysis, consequence domains are treated as downstream outcomes associated with sustained *Ikigai* or with the continued personal integration of meaningful roles, relationships and purposes, rather than as defining elements of the concept itself.

**TABLE 4:** Consequences of *Ikigai* identified in the included studies.

Consequence domain	Description	Representative evidence (Study ID)
Emotional stability and psychological balance	Lower depressive symptoms, reduced suicide risk and improved mental well‐being.	Korean evidence: Not explicitly identifiable in included Korean studies (A9, A11, A12). Contextual evidence: A1, A3, A4, A5
Active health orientation and functional preservation	Greater engagement in health‐promoting behaviours and lower risk of functional decline.	Korean evidence: Not explicitly identifiable in included Korean studies (A9, A11, A12). Contextual evidence: A3, A10
Life fulfilment and satisfaction	Enhanced life satisfaction and everyday contentment. In the Korean evidence, fulfilment is often expressed through ordinary routines and relational continuity.	Korean evidence: A9, A12 Contextual evidence: A5, A7
Community integration and social engagement	Sustained or increased participation in community and social networks.	Korean evidence: A9 Contextual evidence: A2, A8

*Note:* Consequences represent downstream outcomes reported as associated with *Ikigai* or closely related concepts of meaning and purpose in the included literature. They are not treated as defining attributes of *Ikigai* itself. For some consequence domains, Korean evidence was not explicitly identifiable; in such cases, contextual evidence is reported for boundary clarification and should not be interpreted as evidence specific to Korea.

#### 3.5.1 Emotional Stability and Psychological Balance

Studies reported associations between higher *Ikigai* (or related meaning constructs) and lower depressive symptoms, reduced suicide risk and improved mental well‐being. In the included set, this domain was informed mainly by broader comparative literature.

#### 3.5.2 Active Health Orientation and Functional Preservation

Studies reported associations between *Ikigai* and greater engagement in health‐promoting behaviours, alongside a lower risk of functional decline. In the included set, evidence for this domain came mainly from non‐Korean or adjacent‐construct studies.

#### 3.5.3 Life Fulfilment and Satisfaction

Studies described life satisfaction and everyday contentment as outcomes associated with meaning‐related expressions. Korean‐context evidence indicates that fulfilment may be expressed through ordinary routines and relational continuity. This is consistent with the interpretation that Korean‐relevant literature does not present meaning solely as an abstract ideal, but as something sustained and enacted through everyday relational life.

#### 3.5.4 Community Integration and Social Engagement

Studies described sustained or increased participation in community and social networks as an outcome associated with related concepts of meaning and purpose.

### 3.6 Related Concepts and Surrogate Terms

In the Korean context, several surrogate terms are used to approximate—but not fully replicate—the meaning of *Ikigai*. These expressions reflect culturally grounded ways of describing purpose, value and meaning in later life and help clarify the conceptual boundaries between *Ikigai* and related Korean‐language constructs (Choi et al. 2005; Lee and Hong 2017).

In the present synthesis, these Korean‐language terms were not treated as interchangeable labels. *Salm‐ui uimi* was used to denote a broad existential interpretation of life meaning, whereas *saeng‐ui boram* captured a more retrospective sense of worth, reward and relational accomplishment. *Jaa‐silhyeon* and *sal‐uiji* were treated as adjacent concepts because they emphasise personal growth and will to continue living, respectively, rather than the full source‐and‐sense structure of *Ikigai*. This distinction helped clarify why no single Korean term was used as a complete equivalent of *Ikigai*.

#### 3.6.1 *Salm‐ui uimi* (삶의 의미; meaning of life)

In Korean usage, this term refers to a broad existential construct centred on the cognitive and reflective interpretation of life meaning. It overlaps with *Ikigai* in existential meaning, but does not fully capture the more lived, embodied and everyday vitality associated with *Ikigai* (Choi et al. 2005; Lee and Hong 2017).

#### 3.6.2 *Saeng‐ui boram* (생의 보람; life's reward)

In Korean usage, this term refers to a sense of life's worth or reward, often understood retrospectively through effort, contribution, endurance and relational accomplishment across the life course. It overlaps with worth‐ and fulfilment‐related aspects of *Ikigai*, but is narrower and more retrospective in emphasis than *Ikigai* as a lived sense that life is worth living (Choi et al. 2005; Lee and Hong 2017).

#### 3.6.3 *Jaa‐silhyeon* (자아실현; self‐actualisation)

In Korean discussions, this term refers to a psychological construct centred on realising one's potential and personal growth. It is better understood as an adjacent construct rather than a direct surrogate, because it may be more individual‐focused than the relational and intergenerational dimensions often emphasised in later‐life *Ikigai*‐related meaning (Lee and Hong 2017).

#### 3.6.4 *Sal‐uiji* (살 의지; will to live)

In Korean nursing and gerontological discourse, this term refers to a fundamental existential drive or orientation towards continuing life, especially in the context of illness, frailty or later‐life adaptation. It parallels the sustaining function of *Ikigai*, but is more explicitly tied to survival, perseverance and willingness to continue living (Choi et al. 2005; Lee and Hong 2017).

Together, these terms indicate that Korean‐language discussions of later‐life meaning draw on multiple dimensions—existential meaning, perceived worth, personal growth and vitality—that overlap with, but are not identical to, *Ikigai*. Table S3 expands this comparison by distinguishing *Ikigai* from Korean‐language surrogate expressions and adjacent constructs, including meaning in life, purpose in life, self‐actualisation and well‐being. It highlights both shared elements and features more specifically emphasised in the *Ikigai* framework, including valued sources, everyday vitality, future orientation and a sense of life's worth.

### 3.7 Conceptual Model

**FIGURE 2:**
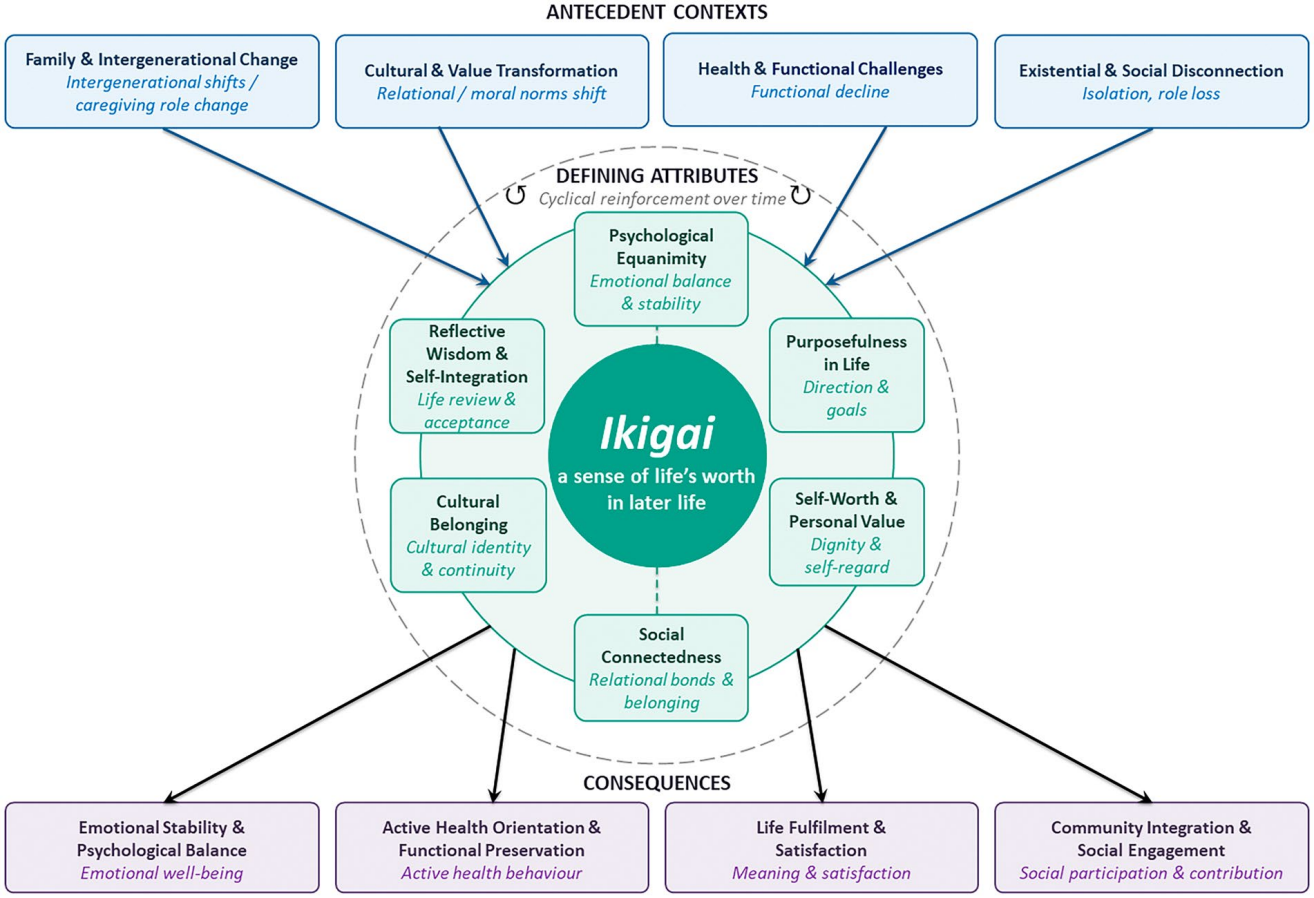
Provisional conceptual model of Ikigai in later life: antecedent contexts, defining attributes of the sense that life is worth living and reported consequences (evidence relevant to older people in Korea). This model synthesises patterns identified in the included literature and is intended to illustrate conceptual relationships rather than causal pathways. ‘Antecedent contexts’ represent conditions that may prompt re‐evaluation, maintenance, reconstruction or renegotiation of Ikigai in later life. In this review, Ikigai is interpreted as involving both valued sources of meaning and a sense of life's worth; the defining attributes shown here represent experiential qualities of that lived sense rather than external sources themselves. Although interpersonal connectedness is particularly salient in Korean‐relevant evidence, it is presented here as one important dimension of Ikigai rather than as its sole or defining basis. Blue arrows link antecedent contexts to the defining attributes, and black arrows link the attributes to reported consequences; both indicate theorised directional associations reported across studies, and do not imply causality. The dashed circle and looped symbols indicate cyclical reinforcement over time among attributes and consequences.

The integrated findings were synthesised into a conceptual model of *Ikigai* (Figure 2), illustrating the relationships among antecedent contexts, defining attributes and consequences (Tables 2, 3, 4). In this article, “process” refers to the conceptual linkages through which older people encounter changing life contexts, interpret or reinterpret valued sources of meaning and sustain or reconstruct the sense that life is worth living. It does not imply a historical change in the definition of *Ikigai*.

At the upper level of the model, antecedent contexts represent sociocultural and psychosocial conditions described as situations in which people may re‐evaluate, maintain, reconstruct or renegotiate *Ikigai* (Table 3). These include family and intergenerational change, cultural and value transformation, health and functional challenges, and existential and social disconnection.

The defining attributes describe how *Ikigai* is articulated as a sense that life is worth living, integrating purpose, self‐worth, relationships, cultural belonging, and reflective integration (Table 2). Where Korean evidence was explicitly identifiable (A9, A11, A12), relational responsibility and intergenerational continuity were particularly salient. The Korean‐relevant literature suggests that these relational sources are not merely listed as external determinants but are taken up, personally integrated and carried into later‐life experience as value, continuity and meaning. At the same time, interpersonal connectedness is not presented here as the sole or defining basis of *Ikigai*; valued sources may also include individual roles, activities or responsibilities that carry personal significance independently of social relationships. Where Korean evidence was not explicitly identifiable, contextual evidence is presented for boundary clarification as described in the notes to Table 2.

At the lower level, consequences illustrate downstream outcomes reported as associated with *Ikigai* or closely related concepts of meaning and purpose in the included literature (Table 4). These include emotional stability and psychological balance, active health orientation and functional preservation, life fulfilment and satisfaction, and community integration and social engagement. Because participation and relationships may also shape subsequent contexts, the model allows for potential feedback links consistent with a cyclical representation.

This framework provides a working foundation for gerontological nursing assessment and intervention planning, aligning conceptual attributes with practice‐oriented indicators and illustrative interventions (Table S2).

### 3.8 Exemplars of *Ikigai* in Later Life

Consistent with Rodgers' fifth step, exemplars were identified to illustrate how *Ikigai* was expressed in the included literature as more than a list of valued sources. In this review, exemplars were used to show how roles, relationships, responsibilities or everyday practices became taken up and personally integrated as a sense of life's worth in later life. Additional exemplar mapping is provided in Table S4.

In the Korean‐context evidence, exemplar patterns were most clearly evident in studies showing that meaning was organised around family continuity, relational responsibility and the creation of value within everyday life. For example, the Korean qualitative meta‐integration (A9) did not portray meaning in later life simply as the presence of supportive relationships, but as an ongoing experience in which family‐based life change, meaning in later life and the creation of meaning were woven together in everyday living. Likewise, the Korean scale‐development study (A11) suggested that later‐life meaning was articulated through value of life, source of life and will to live, indicating that *Ikigai*‐related meaning was not reducible to external roles alone but was also experienced as continued personal significance and vitality. The Korean correlational study (A12) further showed that family relations and self‐transcendent value were closely linked with successful ageing, illustrating how relational sources may be carried into a broader sense of worth and fulfilment in later life.

A contextual comparison exemplar was also evident in the qualitative caregiving study (A13), in which *Ikigai* was described as life experiences and positive feelings that made life worthwhile, while caregiving roles simultaneously shaped self‐understanding. This exemplar is relevant because it illustrates the conceptual distinction central to the present review: valued roles and responsibilities may function as sources of *Ikigai*, but the concept also involves the sense that these sources make life worth living.

These exemplars therefore support the present interpretation that, in literature relevant to older people in Korea, *Ikigai* is most usefully understood not as a fixed object or single activity, but as a dynamic meaning process through which valued sources are personally integrated and sustained as a sense of life's worth.

## 4 Discussion

This evolutionary concept analysis clarified how *Ikigai* is conceptualised in later life and how it may be interpreted within literature relevant to older people in Korea. By distinguishing Korean‐context evidence from contextual evidence used for boundary clarification, this review positions *Ikigai* not as a static trait but as a relational and dynamic meaning process shaped by life transitions. The findings suggest that *Ikigai* in later life reflects an ongoing negotiation between personal meaning, social roles and culturally embedded values. Importantly, the Korean‐relevant literature did not simply identify relational sources of meaning; rather, it showed how family roles, intergenerational ties and everyday responsibilities could be taken up and personally integrated as part of a sense of life's worth in later life.

### 4.1 Defining Attributes: Relationally Grounded Meaning

Across studies, psychological equanimity, purposefulness in life, self‐worth and personal value, social connectedness, cultural belonging, and reflective wisdom and self‐integration emerged as defining attributes. Rather than functioning as discrete psychological states, these attributes appear to operate interactively. In the Korean evidence, purpose and self‐worth were articulated through value of life, will to live, family roles, intergenerational continuity and socially recognised contributions (A9, A11, A12; Kim and Kil 2019; Lee and Hong 2017; Chong et al. 2012). This does not mean that *Ikigai* in Korean later life is reducible to family or relationship sources alone. Rather, the Korean‐relevant literature suggests that relational sources become meaningful through personal interpretation, integration and continuity across lived experience (A9, A11, A12). In this sense, *Ikigai* in this context is not solely an individual sense of life fulfilment but a lived and relationally mediated meaning structure embedded in moral and sociocultural frameworks (A9, A11, A12; Kim and Kil 2019; Lee and Hong 2017; Chong et al. 2012).

It is important to note that interpersonal connectedness, while particularly salient in Korean‐relevant evidence, is not presented as the sole or defining basis of *Ikigai*; valued sources may also include individual roles, activities or responsibilities that carry personal significance independent of social relationships.

The included literature did not consistently label psychological equanimity as resilience. Instead, emotional steadiness was described in terms of acceptance, role continuity and the maintenance of relational responsibility (A1, A4, A5). This distinction is important because *Ikigai* should not be conflated with generic psychological constructs. The present synthesis therefore conceptualises equanimity as an experiential quality associated with a sustained sense of life's worth, rather than as a downstream mental health outcome (A1, A4, A5; Kim and Kil 2019; Watanabe et al. 2024; Aviad and Cohen‐Louck 2021; Dewitte et al. 2022).

### 4.2 Antecedent Contexts: Meaning Renegotiation Rather Than Causal Triggers

The four antecedent contexts—family and intergenerational change, cultural and value transformation, health and functional challenges, and existential and social disconnection—should not be interpreted as direct causes of *Ikigai*. Rather, they represent conditions in which *Ikigai* may be re‐evaluated, disrupted, sustained, reconstructed or renegotiated. Accordingly, antecedent contexts are interpreted as conditions for the renegotiation of meaning rather than as implying that *Ikigai* is exclusive to older adults (Statistics Korea 2023; Kim and Kil 2019; Fukuzawa et al. 2019).

Although these transitions are not unique to Korea, the Korean evidence highlights the importance of family obligation, reciprocity and culturally embedded norms in shaping how older people interpret such changes (A9, A12). Accordingly, the Korean emphasis lies not in the occurrence of these transitions themselves, but in the relational and culturally situated ways in which they are understood and translated into value, continuity and life worth (A9, A12; Chong et al. 2012; Kim and Kil 2019; Choi et al. 2005).

### 4.3 Consequences: Downstream Well‐Being Across Domains

The included studies linked *Ikigai* with outcomes across emotional, behavioural, existential and social domains. Stronger *Ikigai* was associated with emotional stability, health‐oriented behaviour, life satisfaction and sustained social engagement. However, consistent with the evolutionary approach, these consequences should be understood as downstream outcomes associated with the presence of *Ikigai* or closely related concepts of meaning and purpose, rather than as defining components of the concept itself. This distinction is particularly important in the present review because several qualities that appear close to *Ikigai*—such as emotional steadiness or life satisfaction—may be better interpreted as outcomes of sustained meaning rather than as the concept itself (Watanabe et al. 2024; Zhu et al. 2023; Mori et al. 2017; Fukuzawa and Sugawara 2023; Aydın et al. 2020). Compared with broader constructs such as meaning in life or purpose in life, *Ikigai* was distinguished in this review by its combined emphasis on valued sources, everyday vitality, future orientation and a sense of life's worth. Within the present synthesis, “valued sources” was reflected in the purposefulness in life, self‐worth and personal value, social connectedness and cultural belonging attributes; “everyday vitality” was reflected primarily in the value of life and will to live components of the Korean scale study (A11) and in qualitative descriptions of daily meaning‐making (A6, A9); “future orientation” was reflected in articulated goals, roles and intentions across A3, A6 and A11; and “a sense of life's worth” was reflected in the overarching framing of *Ikigai* as more than the presence of valued sources (A9, A11, A13). The simultaneous coupling of valued sources—what gives life meaning—with everyday vitality—the felt sense of being alive in routine activities—therefore helps distinguish *Ikigai* from meaning‐ and purpose‐centred constructs, which more often emphasise cognitive significance or long‐range goal direction in isolation. However, these boundaries remain provisional because several included studies examined adjacent meaning constructs rather than *Ikigai* directly. Table S3 provides an expanded comparison of *Ikigai* with meaning in life, purpose in life, self‐actualisation and well‐being.

Korean evidence directly supported life fulfilment, family‐connected meaning and social participation (A9, A12). For other consequence domains, particularly emotional stability and health preservation, contextual comparison studies provided supporting evidence (A1, A3, A4, A5, A10). These distinctions were made explicit to avoid overstating Korea‐specific claims. (Kim and Kil 2019; Chong et al. 2012; Lee and Hong 2017).

### 4.4 Conceptual Integration: *Ikigai* as a Cyclical Process

The proposed conceptual model depicts *Ikigai* as a cyclical process that links contextual transitions, valued sources of meaning, experiential attributes and downstream well‐being outcomes. Life events may destabilise meaning; through reflective integration, relational engagement and purpose reorientation, older people may reinterpret or sustain valued roles, relationships and responsibilities, thereby reconstructing a sense of life's worth. Consequences such as emotional steadiness and community participation may in turn reinforce this sense of *Ikigai*, supporting a dynamic feedback loop (Rodgers 2000; Park 2010).

The distinction among antecedent contexts, defining attributes and consequences should be understood as an analytic distinction used for concept analysis rather than as a strict separation in lived experience. In particular, the attribute–consequence distinction is intended to support conceptual mapping rather than to imply that experiential qualities and downstream outcomes are independent in practice. These elements may recursively reinforce one another over time; therefore, the model should be read as a provisional analytic framework for understanding *Ikigai* as a dynamic and mutually reinforcing meaning process. As discussed above and summarised in Table S3, *Ikigai* shares important elements with meaning in life, purpose in life, self‐actualisation and well‐being, but is provisionally distinguished by the combined source‐and‐sense structure emphasised in the *Ikigai* framework. These boundaries remain provisional because several included studies examined adjacent meaning constructs rather than *Ikigai* directly.

This model does not posit that *Ikigai* is culturally exclusive. Rather, it illustrates how, within literature relevant to older people in Korea, meaning reconstruction is frequently articulated through relational continuity and culturally embedded moral frameworks. By explicitly separating Korean‐context evidence from contextual comparison evidence, the model avoids conflating cross‐cultural generalities with Korea‐specific claims (Leininger and McFarland 2006).

### 4.5 Limitations

This evolutionary concept analysis has several limitations. First, the evidence base was relatively small and heterogeneous, comprising 13 studies, including 10 conducted outside Korea and three conducted in Korea. In addition, several included studies examined related constructs such as meaning in life or purpose in life rather than *Ikigai* explicitly. Although Rodgers' (2000) evolutionary approach supports the inclusion of related and surrogate concepts to clarify conceptual boundaries, this also requires caution in interpretation. Limitations inherent to Rodgers' evolutionary concept analysis should also be acknowledged. The synthesis relied on secondary literature rather than primary data and was shaped by the methodological, conceptual and contextual diversity of the available studies. To reduce over‐interpretation, we distinguished Korean‐context evidence from contextual comparison evidence and limited Korea‐specific interpretations to findings explicitly identifiable in the Korean studies, while using broader comparative literature to clarify attributes and conceptual boundaries (JBI 2020; Rodgers 2000; Whittemore and Knafl 2005). Because this review focused on contemporary concept usage rather than the historical evolution of *Ikigai*, the findings should not be interpreted as tracing chronological changes in the concept over time. Korean‐context interpretation was therefore treated as a contextual and supplementary analytic layer rather than as the basis for establishing a distinctly Korean conceptual structure of *Ikigai*. Furthermore, within the included Korean studies, evidence for the self‐worth and personal value and purposefulness in life attributes was drawn primarily from a single scale‐development study (A11; Lee and Hong 2017), while social connectedness, cultural belonging, and reflective wisdom and self‐integration were supported by the Korean qualitative meta‐integration (A9) and the Korean correlational survey (A12). This uneven distribution of Korean evidence across attributes reflects the limited and methodologically heterogeneous Korean empirical base on *Ikigai* and related constructs and should be kept in mind when interpreting attribute‐level Korean salience in Tables 2, 3, 4.

Second, the search was restricted to peer‐reviewed publications in English and Korean. Relevant grey literature, non‐indexed regional publications or studies published in other languages may therefore have been missed. Third, cross‐cultural transferability remains constrained because *Ikigai* has no exact Korean equivalent. Mapping *Ikigai* onto Korean‐language surrogate terms such as salm‐ui uimi (삶의 의미), saeng‐ui boram (생의 보람), jaa‐silhyeon (자아실현) and sal‐uiji (살 의지) may have introduced semantic variation and conceptual drift, particularly when these terms were used to illuminate conceptual boundaries rather than to indicate direct equivalence (Choi et al. 2005; Lee and Hong 2017).

Fourth, most included quantitative studies used cross‐sectional and self‐report designs, which limit causal inference and may be vulnerable to reporting bias. Measurement equivalence across cultural settings also remains uncertain, especially because validated instruments that directly capture *Ikigai* in Korean later‐life contexts remain limited (Lee and Hong 2017). In addition, some domains identified in this review—particularly psychological equanimity, health and functional challenges, active health orientation and existential or social disconnection—were supported primarily by contextual comparison studies rather than explicitly identifiable Korean evidence. Accordingly, these domains should not be interpreted as established Korea‐specific findings, but rather as conceptually relevant comparative evidence.

Finally, although methodological and conceptual quality were appraised using criteria adapted from the JBI Critical Appraisal Tools and Whittemore and Knafl's integrative review framework, a formal assessment of publication bias was not feasible given the diversity of designs and the concept‐analytic purpose of the review (JBI 2020; Whittemore and Knafl 2005). For these reasons, the present synthesis should be understood as a theoretically grounded, contextually informed and still provisional interpretation of how *Ikigai* is manifested in literature relevant to older people in Korea, rather than as a definitive statement of a uniquely Korean form of *Ikigai*. Future research should expand Korean empirical work on *Ikigai*, employ longitudinal, qualitative and participatory designs, refine and validate Korean‐language measurement approaches, and examine more directly how valued relational sources are taken up and personally integrated within later‐life experience in Korea.

### 4.6 Implications for Gerontological Nursing

For nursing practice, the findings underscore the importance of assessing meaning in relational and culturally grounded terms. Assessment questions that explore valued roles, intergenerational ties, and perceived dignity may help identify sources of *Ikigai*. Drawing on the defining attributes identified in this review, gerontological nurses may invite older people into a conversation about meaning through open‐ended prompts such as “What gives you a reason to get up in the morning?”, “In what situations do you feel most valued and most like yourself?”, and “When you look back on your life, what lessons or strengths stand out?”—assessment prompts aligned, respectively, with purposefulness, self‐worth and reflective wisdom and self‐integration (Table S2).

Because *Ikigai* may not always be explicitly verbalised by older people, nursing assessment should not rely solely on direct questions. Nurses may also attend to clinically assessable indicators that are observable in everyday care encounters—for example, the articulation of valued goals or roles, regular social contact and participation in meaningful activities, continuity of culturally familiar routines and rituals, and life‐review or generativity themes during conversation (Table S2). These observational cues are particularly important when cognitive change, illness or reticence make direct articulation difficult, and they support assessment of *Ikigai* expressed indirectly through routines, roles and relationships. Interventions such as life review, culturally responsive care planning and support for role continuity can then facilitate meaning reconstruction during transitions such as illness, bereavement or retirement (Chochinov 2007; Reed 2008; Haugan 2014; WHO 2024).

Technology‐enabled approaches may also complement *Ikigai*‐promoting care, particularly when mobility limitations, geographical dispersion or social isolation restrict in‐person engagement. For example, telehealth‐based coaching, moderated online peer groups and digital life‐review or storytelling platforms may help sustain social connectedness and continuity of valued roles; however, digital exclusion, usability barriers and privacy considerations should be anticipated in older populations. Emerging evidence on socially assistive technologies indicates their potential to facilitate engagement and reduce loneliness, but culturally sensitive co‐design and evaluation in Korean settings are needed (Randall et al. 2022; WHO 2024). These technology‐enabled approaches should complement, rather than replace, relational nursing care.

*Ikigai* should not be framed as an expectation that older people must remain productive or resilient. Accordingly, the active health orientation consequence should be interpreted as supported participation in health and function—not as an individual obligation. Meaning‐centred nursing care should be paired with attention to structural and environmental barriers (e.g., poverty, transportation, digital exclusion) that constrain older people's opportunities to sustain *Ikigai*. Instead, *Ikigai* may be understood as a clinically relevant lens through which nurses can recognise and support coping grounded in meaning, relational identity and culturally embedded strengths (Park 2010).

### 4.7 Implications for Research, Education, Policy and Sustainable Care Environments

Future research should expand Korean empirical work on *Ikigai* and related Korean‐language constructs using qualitative, longitudinal and participatory designs. Such studies are needed to examine how older people describe valued sources of meaning in their own words, how these sources are sustained or renegotiated during later‐life transitions, and how they relate to health, care and social participation over time (A9, A11, A12; Choi et al. 2005; Lee and Hong 2017). Measurement research should also refine and validate Korean‐language approaches that distinguish valued sources of *Ikigai* from the experiential sense of life's worth (A11; Lee and Hong 2017).

For nursing education, the findings suggest the need to prepare nurses to assess meaning, dignity, role continuity and relational identity as part of holistic gerontological care. Educational programmes can incorporate case‐based learning, reflective dialogue and culturally responsive communication strategies so that nurses can recognise both explicitly stated and indirectly expressed sources of meaning (Chochinov 2007; Reed 2008; Haugan 2014).

For policy and service design, the findings support age‐friendly care and community environments that reduce isolation, enable safe participation and sustain everyday relational life. Policies that support accessible transport, community participation, digital inclusion and continuity of valued social roles may create broader environmental conditions for sustaining meaning in later life (WHO 2024). Importantly, the sources of *Ikigai* described in the included literature frequently took the form of relational and everyday activities, including family care, intergenerational ties, community participation and continuity of valued roles (A9, A12; Kim and Kil 2019; Chong et al. 2012). Such activities may support meaning in later life while aligning with sustainable, community‐based approaches to care. *Ikigai*‐informed gerontological nursing therefore connects with planetary health in a substantive rather than only rhetorical sense: sustainable care systems that preserve relational continuity, reduce unnecessary fragmentation of care and enable older people to remain embedded in their communities may support both later‐life meaning and broader environmental goals (Leininger and McFarland 2006; WHO 2024). Implications for research, education, practice and policy should accordingly be framed at concentric scales, in which individual *Ikigai* is shaped by, and in turn helps to shape, the relational, community and environmental conditions of later‐life care.

## 5 Conclusion

This evolutionary concept analysis synthesised how *Ikigai* and closely related concepts of meaning and purpose are described in literature relevant to older people in Korea and considered their implications for gerontological nursing. The findings portray *Ikigai* as a dynamic and relational meaning process through which older people may sustain purpose, self‐worth, connection and psychological steadiness during later‐life transitions. Rather than positioning *Ikigai* as culturally exclusive, this analysis suggests that Korean‐context evidence highlights the salience of interdependence, relational responsibility and continuity across generations as common ways of articulating what makes life worth living. More specifically, the Korean‐relevant literature suggests that *Ikigai* or closely related meaning structures are not presented simply as lists of valued sources, but as processes through which family roles, responsibilities and relational continuity are personally interpreted and integrated as a sense of life's worth.

The conceptual model developed in this study links antecedent contexts, defining attributes and downstream consequences. Defining attributes describe how *Ikigai* is experienced and sustained, including psychological equanimity, purposefulness, self‐worth and personal value, social connectedness, cultural belonging, and reflective wisdom and self‐integration. Consequences reported in the included literature include emotional stability, health‐oriented engagement and functional preservation, everyday fulfilment and social participation. Evidence mapping in Tables 2, 3, 4 makes explicit where Korean evidence was explicitly identifiable and where contextual evidence was used for boundary clarification.

For nursing theory and practice, these findings support a culturally responsive framework for meaning‐centred care. Nurses can assess and strengthen sources of meaning through personalised conversations about valued roles, relationships and daily routines and can integrate reflective dialogue and life‐review approaches to support continuity of identity. Interventions that reinforce autonomy in everyday decisions, social connection and continuity of valued roles may assist older people in sustaining or renegotiating *Ikigai* during transitions. At the system level, supportive care and community environments that reduce isolation, enable safe participation and sustain everyday relational life may further strengthen meaning in later life. Age‐friendly communities and sustainable care systems that support older people's social participation and relational continuity may also provide broader environmental conditions for sustaining meaning in later life. These implications extend to research, education, practice and policy because individual experiences of *Ikigai* are shaped not only by personal resources, but also by the relational, community and environmental conditions in which later‐life care is organised. Sustainable care environments that reduce isolation, support continuity of relationships and enable meaningful participation can therefore contribute both to later‐life meaning and to broader goals of environmentally responsible gerontological nursing and planetary health.

Further empirical and measurement research is needed to refine this provisional conceptualisation across diverse Korean later‐life and care contexts and to examine how nursing, community and policy interventions can support meaning, participation and sustainable care environments over time.

## Author Contributions

HeeKyung Chang conceptualised the study and provided overall scholarly supervision. JuHee Seo designed the study, conducted the literature search, coordinated data analysis and drafted the original manuscript. Minji Park and Youngjoo Do contributed to data extraction, methodological refinement and critical revision of the manuscript. All authors independently conducted the quality appraisal of the included studies and participated in data verification and interpretation through consensus discussions. All authors reviewed and approved the final manuscript and agree to be accountable for all aspects of the work.

## Funding

This research was supported by the National Research Foundation of Korea (NRF) and funded by the Ministry of Science and ICT, Republic of Korea (No. RS‐2025‐00558940).

## Ethics Statement

Ethical approval was not required for this study, as it was a concept analysis based solely on previously published literature.

## Conflicts of Interest

The authors declare no conflicts of interest.

## Supporting information


**Table S1:** Detailed study characteristics and quality appraisal (A1–A13).
**Table S2:** Nursing assessment questions, clinically assessable indicators and illustrative interventions aligned with defining attributes of *Ikigai*.
**Table S3:** Comparison of *Ikigai* and related Korean‐language surrogate or adjacent terms.
**Table S4:** Exemplars of *Ikigai* in Later Life Identified in the Included Studies.

## Data Availability

Data sharing is not applicable to this article as no datasets were generated or analysed during the current study.
