# Supplementary material for: A Context‐Informed Evolutionary Concept Analysis of Ikigai in Later Life: Evidence Relevant to Older People in Korea
Source: Int J Older People Nurs. 2026 Jul 3;21(4):e70092. doi: 10.1111/opn.70092 (PMC13332341; doi:10.1111/opn.70092)
Supplement: Supplementary file 1 — Table S1: Detailed study characteristics and quality appraisal (A1–A13). Table S2: Nursing assessment questions, clinically assessable indicators and illustrative interventions aligned with defining attributes of Ikigai. Table S3: Comparison of Ikigai and related Korean‐language surrogate or adjacent terms. Table S4: Exemplars of Ikigai in Later Life Identified in the Included Studies. [file OPN-21-e70092-s001.docx]

**Supplementary File 1. Detailed study characteristics and quality appraisal (A1–A13)**

| **ID** | **Study population / sample** | **Design / methods** | **Target construct / term used** | **Operationalisation / Measurement** | **Key findings relevant to concept analysis** | **Korean-context relevance** | **Aim** | **Design** | **Data** | **Korean relevance** | **Concept contribution** | **Overall** |
| --- | --- | --- | --- | --- | --- | --- | --- | --- | --- | --- | --- | --- |
| A1 | Watanabe, Sato, & Kondo (2024) Japan; mountainous/countryside areas Analytic n=6,978 from 7,973 survey responses; adults aged ≥40 years | Quantitative cross-sectional questionnaire survey | *Ikigai* (sense that life is worth living) | Single-item *Ikigai* question + WHO-5 + frequency of going out | Reduced going out during COVID-19 was associated with lower *Ikigai* and worse mental health; supports social connectedness and emotional steadiness as relevant conceptual elements | Indirect comparison only | 2 | 2 | 2 | 1 | 2 | 9/10  (High–moderate) |
| A2 | Fukuzawa & Sugawara (2023) Japan n=418 adults aged ≥75 years | Quantitative cross-sectional survey | *Ikigai* + life satisfaction | Measures of loneliness, social support, social participation, *Ikigai*, and life satisfaction | Loneliness related negatively to *Ikigai*; social support/participation buffered the association; highlights social role and social interaction as central to *Ikigai* | Indirect comparison only | 2 | 2 | 2 | 1 | 2 | 9/10  (High–moderate) |
| A3 | Zhu, Zhao, Zhu, Cheng, Zhang, & Kong (2023) China Total N=992; late-onset depression n=496, healthy controls n=496; age 60–73 years | Quantitative cross-sectional case–control with mediation modelling | Meaning in life (related construct) | HPLP-IIR, MLQ-C, HAMD, IL-6 | Meaning in life partially mediated the relationship between health-promoting lifestyle and depression severity; informs health-oriented and motivational consequences/antecedents rather than *Ikigai* directly | Indirect comparison only | 2 | 2 | 2 | 0 | 1 | 7/10 (Moderate) |
| A4 | Dewitte, Hill, Vandenbulcke, & Dezutter (2022) Belgium; 9 nursing homes n=140 older people with Alzheimer’s disease; 3 annual assessments | Quantitative longitudinal observational study with structured interviews and cross-lagged/latent growth models | Meaning in life (presence of meaning) | Presence of meaning, depressive symptoms, life satisfaction, cognitive functioning | Higher meaning predicted lower later depressive symptoms; meaning could be sustained despite cognitive decline; informs reflective wisdom, self-integration, and lower depressive symptoms as a downstream psychological outcome | Indirect comparison only | 2 | 2 | 2 | 0 | 1 | 7/10 (Moderate) |
| A5 | Aviad & Cohen-Louck (2021) Israel n=195 adults aged 65–100 years | Quantitative cross-sectional survey | Purpose in life / meaning in life (related constructs) | Purpose in Life + locus of control + suicide risk indices | Higher purpose in life related to lower suicide risk and mediated the relation between internal locus of control and suicide risk; supports purposefulness and psychological steadiness | Indirect comparison only | 2 | 2 | 2 | 0 | 1 | 7/10 (Moderate) |
| A6 | Hupkens, Machielse, Derkx, & Abma (2021) Netherlands; community/home-care context n=24 community-dwelling older people receiving home nursing | Qualitative hermeneutic phenomenological study; three interview waves; photo-elicitation | Meaning in life | Semi-structured interviews + photo-elicitation + interpretative phenomenological analysis | Meaning derived from self, others, environment, and living; retaining meaning involved maintaining, adapting, and discovering; informs meaning-making in daily life, social connectedness, and purpose/process orientation | Indirect comparison only | 2 | 2 | 2 | 0 | 1 | 7/10 (Moderate) |
| A7 | Aydın, Işık, & Kahraman (2020) Turkey; nursing homes and community dwellings n=144 (71 nursing-home; 73 community-dwelling older people), age ≥60 | Quantitative cross-sectional study | Meaning in life + spiritual well-being | BSI, Spiritual Well-Being Scale, Meaning in Life Questionnaire | Existing meaning in life was positively related to transcendence and harmony with nature; informs spiritual well-being, culturally grounded meaning, and meaning in life in later life | Indirect comparison only | 2 | 2 | 2 | 0 | 1 | 7/10 (Moderate) |
| A8 | Fukuzawa, Sugawara, & Shimizu (2019) Japan; Kobe community Baseline n=1,068; follow-up n=686; older people aged 60 years and over | Quantitative panel longitudinal study | *Ikigai* | Panel survey of *Ikigai*, human capital, and social capital | Maintaining or increasing social networks moderated the adverse effect of declines in physical health on *Ikigai*; informs relational continuity and community engagement | Indirect comparison only | 2 | 2 | 2 | 1 | 2 | 9/10  (High–moderate) |
| A9 | Kim & Kil (2019) Korea Qualitative interpretive meta-integration of 13 studies on older people in Korea | Qualitative interpretive meta-integration | Meaning of life (Korean context) | Meta-integration of Korean qualitative studies | Themes: change of life based on family, meaning in later life, experience and creation of meaning; central Korean-context source for family-based and culturally grounded meaning | Direct Korean-context evidence | 2 | 2 | 2 | 2 | 2 | 10/10  (High) |
| A10 | Mori, Kaiho, Tomata, Narita, Tanji, Sugiyama, Sugawara, & Tsuji (2017) Japan; Tsurugaya Project n=830 adults aged ≥70 years; 11-year follow-up | Quantitative prospective cohort study | *Ikigai* (sense of life worth living) | Self-reported *Ikigai* categories + LTCI disability follow-up | Stronger *Ikigai* associated with lower risk of incident functional disability; supports health orientation and active living as downstream consequences | Indirect comparison only | 2 | 2 | 2 | 1 | 2 | 9/10  (High–moderate) |
| A11 | Lee & Hong (2017) Korea Qualitative item-generation n=10; psychometric sample n=371 community-dwelling older people | Instrument development / psychometric validation | Meaning in Life Scale for older adults (Korean scale) | Item generation + factor analysis + validity/reliability testing | Three-factor scale: value of life, source of life, will to live; supports self-worth, purposefulness, and will to live in Korean older people | Direct Korean-context evidence | 2 | 2 | 2 | 2 | 2 | 10/10  (High) |
| A12 | Chong, Jo, An, & Jeong (2012) Korea; Busan/Gimhae senior welfare centers Analysed (n = 497; 562 collected; 65 excluded) | Quantitative correlational survey | Meaning of life; family relations; self-transcendence value | Survey of family relations as a source of meaning of life and successful ageing | Family relations were the most important domain of meaning; self-transcendence value and family relations significantly affected successful ageing; strong Korean-context evidence for family connectedness | Direct Korean-context evidence | 2 | 2 | 2 | 2 | 2 | 10/10  (High) |
| A13 | Yamamoto-Mitani & Wallhagen (2002) Japan; caregiving context in Yamaguchi/Tokyo n=26 women family caregivers (13 daughters; 13 daughters-in-law) caring for older parents/parents-in-law living with dementia | Qualitative interview study using constant comparative methodology | *Ikigai* | In-depth interviews on caregiving and self-understanding | *Ikigai* defined as life experiences and/or positive emotions that make life feel worthwhile; pursuit and maintenance of *Ikigai* linked with self-understanding and culturally embedded caregiving roles | Indirect comparison only | 2 | 2 | 2 | 1 | 2 | 9/10  (High–moderate) |

**Abbreviations/scoring.** Aim = clarity of research aims; Design = appropriateness of design/method for concept elucidation; Data = richness of data/analysis for attribute/antecedent/consequence extraction; Korean relevance = relevance to Korean sociocultural context; Concept contribution = explicit contribution to understanding *Ikigai* or closely related constructs. Ratings: 2 = clear/strong, 1 = partial/indirect, 0 = limited or unclear. These ratings were used to support interpretive transparency and were not used as sole grounds for exclusion.

**Supplementary File 2. Nursing assessment questions, clinically assessable indicators, and illustrative interventions aligned with defining attributes of *Ikigai***

| **Defining attribute** | **Supporting studies**  **(provenance; see Notes)** | **Sample nursing assessment questions** | **Clinically assessable indicators** | **Evidence-informed nursing interventions** | **Notes (concept term in source studies)** |
| --- | --- | --- | --- | --- | --- |
| **Psychological Equanimity** | A1, A3, A5 | “When you face difficulties, what helps you stay steady or regain your balance?” “How do you usually cope with stress or uncertainty **in your daily life**?” | Calm and steady affect; acceptance-based coping (reportable); emotional regulation; maintains routines despite stress; reduced distress when supported | Support coping plans; encourage paced routines; mindfulness and relaxation; facilitate access to counselling or peer support; monitor signs of distress and refer when needed | Primarily derived from meaning-in-life literature (meaning/presence of meaning); not all source studies explicitly used the term *Ikigai*. Additional meaning-in-life sources informed indicator wording but are not listed as direct evidence for the attribute label. |
| **Purposefulness in Life** | A3, A6, A11 | “What gives you a reason to get up in the morning?” “What activities or roles make your day feel worthwhile?” | Expresses valued goals/roles; engages in meaningful activities; articulates future-oriented intentions; describes life as worth living or expresses a sustained sense of purpose | Goal-setting with the person; activity adaptation; support role continuity (family/community); connect to volunteering or interest groups; reinforce strengths and autonomy | This attribute was supported directly by Korean evidence from A11 and by contextual evidence from A3 and A6, consistent with Table 2. Other *Ikigai* studies informed broader conceptual boundaries but were not retained as representative evidence for this specific attribute. |
| **Self-Worth and Personal Value** | A11 | “In what situations do you feel most valued and like yourself?” “What is important for you to preserve in daily life?” | Maintains self-respect and personal values; preference articulation; seeks autonomy in daily decisions; preserves identity through roles and narratives | Person-centred communication; shared decision-making; dignity-conserving care; support privacy and preferences; validate identity and life story | This attribute was supported directly by Korean evidence from A11, consistent with Table 2. It was often framed through meaning, value of life, and self-understanding rather than explicit *Ikigai* terminology. |
| **Social Connectedness** | A2, A6, A8, A9, A12 | “Who do you feel close to, and how often do you connect with them?” “What helps you feel connected to others these days?” | Regular social contact; participation in social activities; perceived support; trust and reciprocity; reduced loneliness when connected | Facilitate social participation **adapted to functional ability**; family meetings; community linkage; address barriers to participation (e.g., mobility, transportation, scheduling); support caregiver-family networks | This attribute was supported by Korean evidence from A9 and A12 and by contextual evidence from A2, A6, and A8, consistent with Table 2. |
| **Cultural Belonging** | A7, A9, A12 | “What traditions, values, or relationships make you feel you belong?” “How do your family/community roles shape what feels meaningful?” | Expresses continuity with cultural values; meaning derived from family/intergenerational roles; comfort with culturally familiar routines; sense of belonging within the community | Culturally responsive assessment; incorporate family values in care planning; support culturally meaningful routines/rituals; connect to community resources | This attribute was supported by Korean evidence from A9 and A12 and by contextual evidence from A7, consistent with Table 2. |
| **Reflective Wisdom and Self-Integration** | A4, A6, A9, A12, A13 | “When you look back on your life, what lessons or strengths stand out?” “How do you make sense of changes that come with ageing?” | Life review and meaning-making; balanced appraisal of past experiences; integrates losses and gains; expresses self-transcendence or generativity themes | Guided life-review; narrative therapy elements; support intergenerational storytelling; facilitate legacy projects; promote self-transcendence activities aligned with values | This attribute was supported by Korean evidence from A9 and A12 and by contextual evidence from A4, A6, and A13, consistent with Table 2. |

**Note.** “Supporting studies” indicate which of the 13 included papers (A1–A13) informed each attribute during synthesis; this mapping documents provenance and does not imply exclusivity, direct observability in all settings, or causal direction.

**Supplementary File 3. Comparison of *Ikigai* and related Korean-language surrogate or adjacent terms**

| **Term / construct** | **Literal English gloss** | **Core meaning in Korean usage or adjacent construct** | **Elements shared with *Ikigai*** | **Features more specifically emphasised in *Ikigai*** | **Indicative references** |
| --- | --- | --- | --- | --- | --- |
| ***Ikigai***  **(生きがい)** | A life worth living / what makes life worth living | A dynamic later-life meaning process involving valued sources of worth and a sense of life’s worth | Focal concept in this review | Combines valued sources, everyday vitality, future orientation, and the felt sense that life is worth living | Tanno et al., 2009; Tomioka et al., 2016; Randall et al., 2022, 2023 |
| ***Salm-ui uimi* (삶의 의미)** | Meaning of life | A broad existential construct referring to the cognitive and reflective interpretation of life meaning | Overlaps with *Ikigai* in existential meaning and life significance | Does not fully capture the more lived, embodied, everyday vitality and valued sources associated with *Ikigai* | Choi et al., 2005; Lee & Hong, 2017 |
| ***Saeng-ui boram***  **(생의 보람)** | Life’s reward / worth of life | A sense of life’s worth or reward, often understood retrospectively through effort, contribution, endurance, and relational accomplishment across the life course | Overlaps with worth- and fulfilment-related aspects of *Ikigai* | More retrospective and achievement-oriented than *Ikigai*, which also includes present everyday vitality and future orientation | Choi et al., 2005; Lee & Hong, 2017 |
| ***Jaa-silhyeon* (자아실현)** | Self-actualisation | A psychological construct centred on realising one’s potential and personal growth | Shares the themes of personal growth, agency, and self-development | More individually focused; does not fully capture relational continuity, intergenerational roles, and culturally embedded responsibilities emphasised in later-life *Ikigai* | Lee & Hong, 2017 |
| ***Sal-uiji***  **(살 의지)** | Will to live | A fundamental existential drive or orientation toward continuing life, especially in the context of illness, frailty, or later-life adaptation | Parallels the sustaining function of *Ikigai* and willingness to continue living | More explicitly tied to survival and perseverance, whereas *Ikigai* includes valued sources, meaning, vitality, and life worth | Choi et al., 2005; Lee & Hong, 2017 |
| **Meaning in life** | Meaning of life / life meaning | A broad construct concerning perceived significance, coherence, and meaning in one’s life | Shares existential meaning, perceived life significance, and reflective interpretation | *Ikigai* is not limited to cognitive meaning; it also links concrete sources of worth with an everyday felt sense that life is worth living | Choi et al., 2005; Lee & Hong, 2017; Dewitte et al., 2022 |
| **Purpose in life** | Direction or purpose in life | A construct focused on goals, direction, future orientation, and motivation | Shares direction, goals, future orientation, and motivation to continue living | *Ikigai* may include purpose, but also emphasises concrete sources such as roles, relationships, activities, and culturally embedded responsibilities | Aviad & Cohen-Louck, 2021; Zhu et al., 2023; Lee & Hong, 2017 |
| **Self-actualisation** | Realising one’s potential | A psychological construct focused on personal growth, self-development, and fulfilment of one’s potential | Shares personal growth, agency, and self-development | *Ikigai* is less exclusively individual-focused and may be grounded in relational continuity, intergenerational roles, and contribution to others | Lee & Hong, 2017; Kim & Kil, 2019 |
| **Well-being** | Positive well-being / life satisfaction | A broad outcome-oriented construct including positive affect, life satisfaction, psychological balance, and vitality | Shares positive affect, life satisfaction, psychological balance, and vitality | In this review, well-being was treated mainly as a related outcome, whereas *Ikigai* refers to a meaning process and experiential sense that may support well-being. | Tanno et al., 2009; Tomioka et al., 2016; Watanabe et al., 2024; Aydın et al., 2020 |

**Note.** This table compares *Ikigai* with Korean-language surrogate expressions and adjacent constructs of meaning, purpose, self-development, and well-being. The terms and constructs overlap with *Ikigai* in important ways, but none functions as a complete one-to-one equivalent. The comparison is intended to clarify conceptual boundaries rather than to establish mutually exclusive categories. In this review, *Ikigai* was distinguished by its combined emphasis on valued sources, everyday vitality, future orientation, and a sense of life’s worth.

**Supplementary File 4. Exemplars of *Ikigai* in Later Life Identified in the Included Studies**

| **Study ID** | **Source context** | **Exemplar description** | **Relevance to concept analysis** |
| --- | --- | --- | --- |
| **A9** | Korean qualitative interpretive meta-integration of 13 studies on older people in Korea | Meaning in later life was not described simply as the presence of family or social roles, but as an ongoing experience organised around family-based life change, meaning in later life, and the creation of meaning. | Illustrates a Korean-context exemplar in which family-based and relational sources are taken up and lived as continuing meaning, rather than remaining external conditions only. |
| **A11** | Korean scale-development study with qualitative item generation and psychometric testing | Later-life meaning was structured through value of life, source of life, and will to live. | Shows that Korean later-life meaning includes both valued sources and an internal sense of significance and vitality, supporting the distinction between source and lived sense. |
| **A12** | Korean correlational survey of family relations, meaning of life, self-transcendence value, and successful ageing | Family relations and self-transcendence value were strongly associated with successful ageing, indicating that relational ties may be carried into a broader sense of worth, contribution, and fulfilment in later life. | Functions as a Korean-context exemplar of how relational sources may be internalised as meaningful continuity and later-life worth. |
| **A13** | Japanese qualitative interview study of women caring for older parents or parents-in-law living with dementia | *Ikigai* was described as life experiences and/or positive feelings that make life worthwhile, while caregiving roles simultaneously shaped self-understanding. | Provides a contextual comparison exemplar showing that valued roles may serve as sources of *Ikigai*, while a sense of life’s worth remains a distinct experiential dimension. |

**Note.** Exemplars were identified in accordance with Rodgers’ fifth step to illustrate how *Ikigai* and closely related concepts of meaning and purpose were expressed in the included literature. These exemplars are not intended as exhaustive summaries of each study, but as concise illustrations of how valued roles, relationships, responsibilities, or everyday practices became linked to a sense of life’s worth. Korean-context interpretation was anchored primarily in the Korean studies (A9, A11, A12), while A13 was included as a contextual comparison exemplar.
